# Bridging conventional clinical tests and advanced biomarkers for the early diagnosis of kidney disease progression

**DOI:** 10.12669/pjms.41.11.12785

**Published:** 2025-11

**Authors:** Bakhtawar Farooq, Zahid Habib Qureshi, Muhammad Faisal, Madeeha Shahzad Lodhi

**Affiliations:** 1 Bakhtawar Farooq Institute of Molecular Biology and Biotechnology, Department of Biochemistry, Nishtar Medical University, Multan, Pakistan. The University of Lahore, Lahore, Pakistan; 2 Zahid Habib Qureshi Institute of Molecular Biology and Biotechnology, Department of Physiology, Multan Medical and Dental College, Multan, Pakistan. The University of Lahore, Lahore, Pakistan; 3 Muhammad Faisal Institute of Plant Breeding and Biotechnology, MNS-University of Agriculture, Multan, Pakistan; 4 Madeeha Shahzad Lodhi Institute of Molecular Biology and Biotechnology, The University of Lahore, Lahore, Pakistan

**Keywords:** Asymmetric dimethyl arginine, Beta-2 microglobulins, Chronic kidney disease, Fetuin-A, Klotho, Neutrophil gelatinase-associated lipocalin, Uromodulin

## Abstract

**Objective:**

This study aimed to evaluate and validate the applicability and reliability of seven biomarkers for the early detection of kidney diseases in the Pakistani population.

**Methodology:**

A case-control study involving two hundred participants was conducted at Nishtar and Ibn-e-Siena Hospitals, Multan, from January to December 2022. Seven serum biomarkers, klotho, uromodulin, neutrophil gelatinase-associated lipocalin (NGAL), beta-2 microglobulin, asymmetric dimethyl arginine (ADMA), beta trace protein, and fetuin-A, were exploited, and their levels were measured by enzyme-linked immunosorbent assay (ELISA) kits. Diagnostic performance of these biomarkers was measured through the receiver-operator curve (ROC) via GraphPad Prism. Correlations among biomarkers were assessed by matrix scatter plots, while protein-protein interactions were analyzed using STRING. Data was statistically analyzed using one-way analysis of variance (ANOVA) and the Tukey’s multiple comparison test.

**Results:**

The results demonstrated a significantly reduced eGFR and elevated creatinine levels in chronic kidney disease (CKD) and acute kidney injury (AKI) patients compared to controls. Additionally, all seven diagnostic serum biomarkers were found to be associated with CKD. Uromodulin level was lower in AKI (35ng/ml ±3.8) verses CKD (41.7ng/ml±14.3) patients, while other biomarkers were significantly higher in AKI. Receiver-operator curve (ROC) analysis showed excellent CKD detection, particularly by BTP, NGAL, B2M, ADMA, and uromodulin (AUC>0.99). Furthermore, klotho correlated positively with several biomarkers, while uromodulin had negative correlations, and NGAL showed strong positive correlations overall. STRING results revealed strong interactions among B2M biomarker with uromodulin.

**Conclusions:**

The BTP, ADMA, and Klotho show good potential for improving early diagnosis and differentiation of CKD and AKI, supporting timely and clinical interventions.

## INTRODUCTION

Kidney diseases are recognized as one of the leading causes of human death.^1^ Among these kidney diseases, chronic kidney disease (CKD) and acute kidney injury (AKI) pose a significant challenge globally, with an intense burden in Pakistan. 1-3 AKI is characterized by a sudden decline in kidney function, whereas CKD is a steady loss of kidney function over time.^3^ Persistence of estimated glomerular filtration rate (eGFR) < 60 mL/min/1.73 m^²^ for more than three months confirms CKD, which is a life-threatening disorder requiring intensive nephrologist care.^3^,^4^ CKD stages are categorized by the eGFR level, from normal (Stage-1) to kidney failure (Stage-5).^4^ Serum creatinine and eGFR tests are the gold standard for kidney assessment can be affected by factors such as age, sex, muscle mass and diet, which may compromise accuracy and introduce potential errors.^5^ A regression model cannot fully compensate biological and measurement-related flaws of creatinine, especially those caused by external factors like diet/food (cooked red meat), medication and interfering substances in the blood.^6^ Integrating serum and clinical biomarkers enhances prognosis prediction, may delay dialysis in kidney patients and prevent costly late-stage treatments by enabling early detection.^7^

Beta trace protein (BTP) is a kidney-associated marker expressed in almost all tissues involved in the metabolism of prostaglandins, including the kidney, heart, brain, retina, melanocytes, and male reproductive organs.^8^ Fetuin-A, a glycoprotein from the liver, inhibits vascular calcification, which is linked to increased mortality in CKD.^9^ Another marker, klotho, is a transmembrane protein that can hydrolyze steroid β-glucuronides.^10^ Uromodulin is a glycoprotein primarily produced by the kidneys and secreted into the urine.^11^ Asymmetric dimethyl arginine (ADMA) is another study marker that is an endogenous nitric oxide synthase inhibitor and is known to be strongly and independently correlated with several cardiovascular disorders, pulmonary hypertension, diabetes and kidney disease.^12^

Neutrophil gelatinase-associated lipocalin (NGAL) is one of the important biomarkers expressed in renal tubular cells and is found in urine and plasma at low concentrations.^13^ Beta-2 Microglobulin (B2M) is a small protein on the surface of all nucleated cells in the body.^14^ Identifying highly sensitive and specific biomarkers for the early detection of CKD and their subsequent impact on enhancing patient prognosis represents a significant and unresolved requirement within the medical field and is the basic aim of our study. This study aimed to evaluate and validate the applicability and reliability of seven biomarkers for the early detection of kidney disease in the Pakistani population.

## METHODOLOGY

A case-control study was organized from the outpatient departments of Nishtar and Ibn-e-Siena Hospital Multan, Punjab, Pakistan, from January to December 2022.

### Ethical approval:

The study sample and data collection were conducted in 2022 in Ibn-e-Siena (IRB no.C-18-921 January, 2022) and Nishtar Hospital (IRB no.13323/NMU August, 2022) Multan, Pakistan. Formal approval from University of Lahore (UOL) ethics committee (Ref No. is CRiMM/23/Research/39; October, 2023) was obtained for data analysis. All participants provided written informed consent after receiving a complete explanation of the study objectives and procedures.

### Inclusion criteria:

Participants were eligible for inclusion if they met the following criteria:

- Adults aged ≥18 years.
- Willing and able to provide written informed consent.

*Having one or more of the following comorbid conditions associated with increased risk of* CKD:

a) Diabetes mellitus
b) Hypertension
c) Cardiovascular disease
d) Arthritis
e) Chronic glomerulonephritis

- Healthy controls without these comorbidities, enrolled for comparative analysis.

### Exclusion criteria:

Participants were excluded if they had any of the following:

- Clinically diagnosed acute kidney injury (AKI) or chronic kidney disease (CKD).
- Requirement for dialysis therapy.
- Active malignancies (including uterine fibroids) or undergoing specific oncologic treatments.
- Diagnosed systemic lupus erythematosus (SLE) or rheumatoid arthritis.
- Acute infections, septic shock, or hypotension at the time of recruitment.
- Laboratory-confirmed COVID-19 infection.

The study’s eligibility criteria were shared with consulting nephrologist who reviewed participants records and referred individuals who met the study’s requirements.

### Study population:

The present study included two hundred participants: one hundred thirty with kidney disease and seventy healthy individuals. The sample size of 200 was calculated to estimate a population parameter with 95% confidence interval and 5% absolute precision to achieve 80% power of the test.

### Clinical and laboratory assessment:

The evaluation of kidney disease involved assessing factors, including history, age, weight, gender, smoking and lifestyle habits. After careful assessment of the patient’s vitals, serum creatinine was assayed with the rate-Jaffe reaction on the Siemens analyzer, ADVIA 18, by using calibrators. After creatinine estimated values, eGFR was measured manually through the MDRD equation and cross-checked through an automatic Laboratory Information System machine.

The experiment utilized the Human ELISA Kit (96-well) provided by Bioassay and MyBioSource. These ELISA kits were designed to measure Human klotho (E2781Hu), uromodulin (E4743Hu), Fetuin-A (E1386Hu), beta trace protein (MBS167479), NGAL (E1719Hu), beta-2 microglobulin (MBS700293) and ADMA (E1887Hu) protein levels in samples using sandwich ELISA. ELISA test was performed in research lab with three replicates, a positive control and a negative control. The standard curve range is established as per kit instructions for biomarker quantification within specified concentration limits. The ELISA automatically measures the optical density (OD) of each well using a microplate reader set to 450 nm and exported in Excel file.

### In silico protein-protein interaction study:

Protein-protein interaction network analysis was performed using the online protein interaction retrieval website STRING (http://www.string-db.org/) and visualization of the interaction network was accomplished using Cytoscape software (https://cytoscape.org/).^15^

### Statistical analysis:

SPSS version 25 facilitated data entry and analysis, while quantitative factors were summarized with mean ± SD in Excel. One-way analysis of variance (ANOVA) and the Tukey multiple comparison test were used to analyze data statistically by GraphPad Prism. The areas under the curves (AUCs) compared diagnostic performance, calculated using receiver-operator curve (ROC) analysis in GraphPad Prism. The Youden index was used to determine the optimal cutoff values. The associations among the studied biomarkers were evaluated using Pearson correlation coefficients (r) and corresponding p-values, presented in a matrix scatter plot.

## RESULTS

The present study included two hundred participants: one hundred thirty with kidney disease and seventy healthy individuals. Of 130 patients, 83 (63%) were males and 47 (36%) were females. Similarly, 42 (60%) were males and 28 (40%) were females within the control group (70). The participants’ age was 46 years ± 15 within the patient group. A similar pattern was observed within the control (42±15). Furthermore, the study examined weight, smoking habits, ischemic heart disease, family disease history, hypertension, Type-II diabetes and viral infections. Out of 130 kidney disease participants, 17 were diagnosed with AKI, 113 were CKD [stage-2(10), stage-3 (14), stage-4 (21), stage-5 (68)].

The mean of creatinine was 1.0 mg/dl±0.2 (control), 7.5 mg/dl±4.5 (CKD) and 11 mg/dl±4 (AKI patients), while in CKD stages, the level of creatinine was 2 mg/dl±0.6 (Stage-2), 2.3 mg/dl±0.3 (Stage-3), 3.5 mg/dl±0.5 (Stage-4) and 10 mg/dl±3.2 (Stage-5). The mean eGFR was 74 ml/min/1.73m^2^±19 (control) and 5 ml/min/1.73m^2^±2.8 (AKI patient). For CKD stages, eGFR values were 55 ml/min/1.73m^2^±15 (Stage 2), 35 ml/min/1.73m^2^±4 (Stage-3), 19 ml/min/1.73m^2^±3 (Stage-4) and 7.7 ml/min/1.73m^2^±3 (Stage-5). Therefore, the study reveals key characteristics, eGFR and creatinine values that can help in assessing kidney function and disease severity.

**Fig.1:**
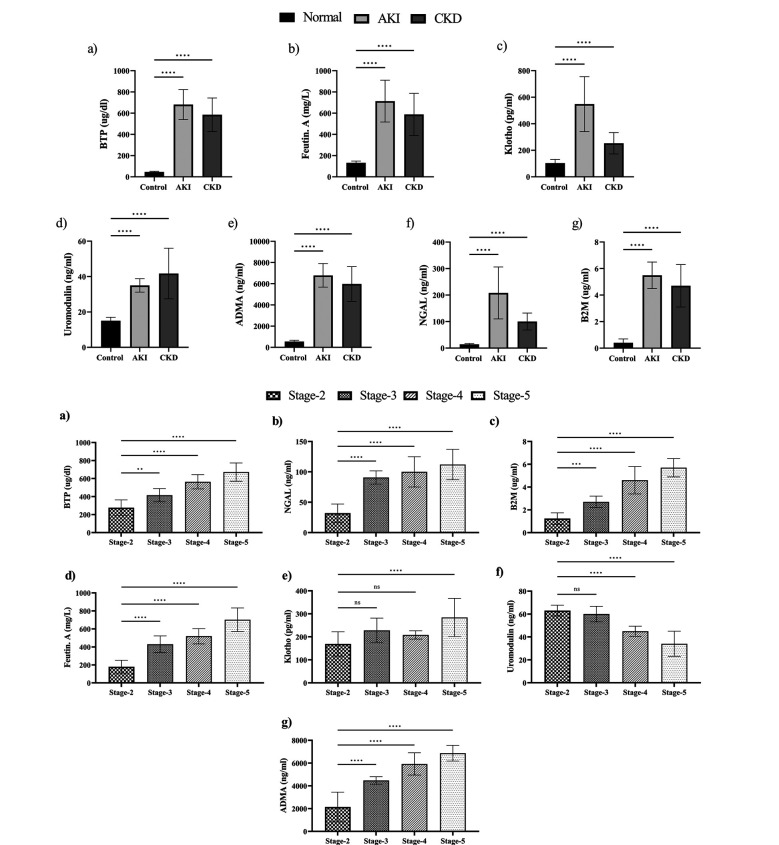
Distribution of biomarkers among AKI, CKD stages and control. Fig-1A shows the biomarkers concentration among AKI, CKD and controls and Fig-1B shows biomarkers concentration among stages of CKD. Data are presented as mean ± SD. One-way ANOVA and Tukey multiple comparison test were used to analyze data statistically. “ns” indicates non-significant, asterisks (*) indicate statistical significance. ****= significance level (P<0.0001).

Seven biomarkers were analyzed, revealing significantly higher concentrations in AKI and CKD patients compared to controls (Fig.1A). The mean plasma concentrations of klotho, uromodulin, NGAL, ADMA, beta trace protein and fetuin-A were significantly higher in AKI and CKD patients compared to their controls (Table-I). All biomarkers were significantly higher in AKI than in CKD patients, except uromodulin that was lower in AKI (35 ng/ml ±3.8) verses CKD (41.7 ng/ml±14.3) patients (Table-I). The diagnostic performance of these biomarkers was assessed using ROC analysis, which showed that the markers had an AUC of 0.6788, 0.9894, 0.9900, 0.9913, 0.9999, 0.9937 and 0.9929 for fetuin-A, klotho, BTP, Uromodulin, ADMA, NGAL and B2M in CKD detection, respectively, as described in Table-II.

**Table-I:** Distribution of biomarkers among AKI, CKD stages and control.

Biomarkers	Control	CKD	CKD Stage				AKI
			Stage-2	Stage-3	Stage-4	Stage-5	
	70	113	10	14	21	68	17
BTP (ug/dl)	46±6.2	585±158	277±86	415 ±74	564±79	672±101	681±144
Fetuin-A (mg/dL)	132±17	588±199	180±72	429±93	519±85	702±131	714±197
Klotho (pg/ml)	103±28	253±80	169±53	228±53	208±18	284±83	548±206
Uromodulin (ng/ml)	15±2.02	41.7±14.3	63±4.7	60±6.8	45±4.43	34±11	35 ±3.8
ADMA (ng/ml)	544±118	5978±1637	2148±1299	4474±334	5925±980	6868±688	6791±1113
NGAL (ng/ml)	14±3.0	100±32.0	32±15.0	90.7 ±11	100±25.0	112±25.0	208±101
B2M (ug/ml)	0.44±1.6	4.7±1.6	1.24 ±0.5	2.7±0.5	4.6±1.26	5.7±0.8	5.49 ±1.04

**Table-II:** Diagnostic performance of biomarkers to assess CKD.

Biomarker	Parameters	AUC	Sensitivity	Specificity	Youden index	95% CI	P value
BTP (ug/dl)	CKD	0.9900	0.942642	0.967403	0.91	0.9718 to 0.9671	<0.0001
	AKI	0.7600	0.702784	0.811279	0.51	0.7800 to 0.9341	<0.0001
Fetuin-A (mg/dL)	CKD	0.6788	0.662014	0.515303	0.18	0.5306 to 0.8270	0.0177
	AKI	0.7482	0.729192	0.638201	0.37	0.7250 to 0.7916	<0.0001
Klotho (pg/ml)	CKD	0.9894	0.924765	0.965613	0.89	0.9739 to 0.9648	<0.0001
	AKI	0.9525	0.756601	0.815789	0.57	0.8618 to 0.9671	<0.0001
Uromodulin (ng/ml)	CKD	0.9913	0.923209	0.961242	0.88	0.9761 to 0.9610	<0.0001
	AKI	0.7233	0.72549	0.807139	0.53	0.7655 to 0.8996	<0.0001
ADMA (ng/ml)	CKD	0.9999	0.894851	0.957316	0.85	0.9400 to 0.9991	<0.0001
	AKI	0.9679	0.92222	0.803178	0.89	0.7218 to 0.8631	<0.0001
NGAL (ng/ml)	CKD	0.9937	0.867085	0.945524	0.81	0.9840 to 0.9942	<0.0001
	AKI	0.9300	0.922222	0.801589	0.73	0.7668 to 0.8601	<0.0001
B2M (ug/ml)	CKD	0.9929	0.85035	0.948381	0.80	0.9827 to 0.9999	<0.0001
	AKI	0.88000	0.709151	0.805556	0.51	0.6778 to 0.8871	<0.0001

***Note:*** AUC = Areas Under the Curves, 95% CI = 95% Confidence Interval.

Moreover, all biomarkers’ mean plasma values in cases exceeded those in controls. When comparing advanced stages of CKD, significant differences were observed among all biomarkers (Fig.1B). Except for uromodulin, there was an increasing trend in biomarker concentrations with advancing CKD stages. A mixed trend of increasing and decreasing concentrations was seen for certain biomarkers across advanced stages. ROC showed excellent CKD detection, predominantly by BTP NGAL, B2M, ADMA and uromodulin (AUC ≥ 0.99). Therefore, tested biomarkers are associated with kidney diseases.

**Fig.2:**
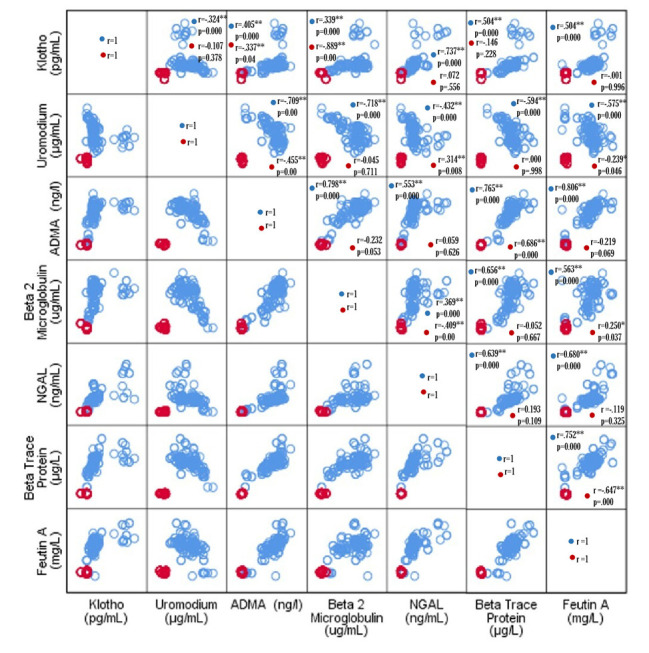
Matrix scatter plot for the correlation study between biomarkers. Blue dots represent cases and red dot represent controls. Data are presented in Pearson correlation coefficients (r) and corresponding p-values.

**Fig.3:**
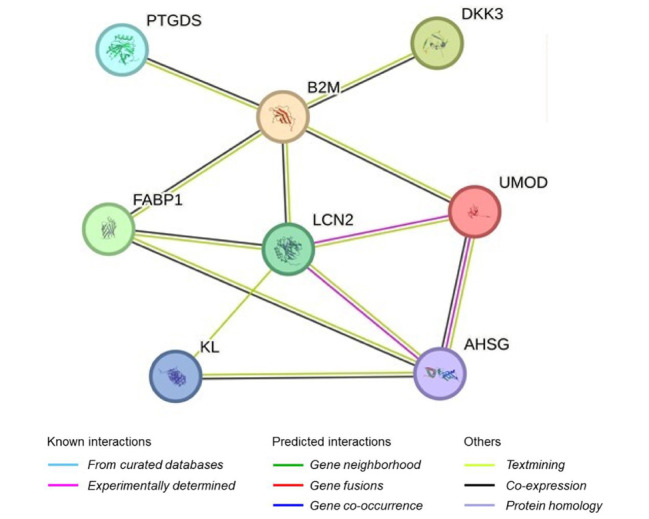
**Interaction network among selected biomarkers.** The network illustrates functional protein-protein associations among the ELISA-quantified biomarkers, highlighting their interconnected roles in disease pathways.

The correlation among seven molecular biomarkers in CKD patients reflects their progression, as shown in a matrix scatter plot (Fig.2). In controls, klotho had a weak to moderate positive correlation with ADMA and a strong negative correlation with beta-2 microglobulin. Uromodulin displayed weak negative correlations with ADMA and fetuin-A and a weak positive correlation with NGAL. In cases, klotho showed weak to moderate positive correlations with uromodulin, AMDA, beta-2 microglobulin, beta trace protein and fetuin-A and a strong correlation with NGAL. Uromodulin demonstrated moderate to strong negative correlations with others, while NGAL exhibited moderate to strong positive correlations overall. Therefore, strong positive correlations suggest interconnections among biomarkers. The pearson’s correlation coefficient (r) values and their corresponding p-values are shown in Fig.2. The STRING database was used to predict the interaction sites of UMOD, KL and B2M proteins and and their association with kidney functioning help in early diagnosis (Fig.3). Each labeled circle in the network represents a specific protein. The lines connecting the nodes represent interactions or associations between these proteins. This search identified the associated proteins including DKK3, LCN2, PTGDS, FABP1, and AHSG proteins. There were eight nodes, each representing all proteins produced including isoforms for each single protein coding gene and 13 edges which indicate both direct predicted functional and physical protein associations or interactions for each gene.

## DISCUSSION

This study evaluated seven serum biomarkers (BTP, fetuin-A, klotho, uromodulin, ADMA, NGAL and B2M) for their potential to distinguish CKD, AKI and healthy controls in a Pakistani cohort (Fig.1). The key findings revealed that BTP, NGAL and ADMA demonstrated outstanding diagnostic performance (AUC >0.99) in CKD while Klotho and Uromodulin also showed high accuracy (Table-II). Correlation analyses highlighted significant interrelationships among these biomarkers, underscoring their promise as a combined panel for early detection and staging of kidney dysfunction (Fig.2).

Our study revealed low eGFR and inconsistent creatinine levels in CKD stages Two and Three, with significant elevation compared to control, which shows inconsistency with traditional methods. Conventional biomarkers for CKD, such as serum creatinine and urea, fail to detect early disease and lack sensitivity and specificity.^5^,^6^ Identifying novel serum biomarkers can bridge this clinical gap by enabling early diagnosis, accurate staging, and improved prognosis of CKD.

This study has shown that the level of beta-trace proteins was significantly higher in CKD and AKI patients compared to controls. Further, the BTP value was higher in AKI compared to CKD (Fig.1). There is a lack of local research on BTP in CKD and AKI patients. However, Thalquotra M et al. reported that the level of BTP increased in Indian CKD patients compared to controls,^16^ which is consistent with our study. Elebidi A et al. identified BTP as a predictive biomarker of CKD and reported that it could be a reliable serum marker for assessing renal dysfunction in CKD patients, serving as an alternative GFR marker.^17^ Further, Leyssens K et al. reported that serum BTP levels were higher in patients developing AKI than in patients not developing AKI, which likely corresponds with a decreased renal clearance.^18^

Previous studies have shown t a reduction in fetuin-A levels in CKD patients.^19^,^20^ Deepa and Sasivathanam found that the fetuin-A levels decrease with decline in renal function and identified significantly lower fetuin-A levels in CKD patients than controls,^19^ which is contrary to our study. Our study found significantly elevated levels of fetuin-A in AKI compared to normal and CKD groups, with an increasing trend in CKD stages and vascular calcification (Fig.1). Several studies have reported that nutritional, environmental and inflammatory responses can affect fetuin-A expression.^21^,^22^ According to Icer MA et al., dietary intake of omega-3 fatty acids can increase fetuin-A concentration, while consumption of dairy products, curcumin, niacin, palmitate, coffee and alcohol decreases fetuin-A levels.^21^

This study also showed klotho values were higher in CKD and AKI compared to the control group (Fig.1A), showing its role in the pathophysiology of kidney injury. Further, Klotho values in AKI patients were found to be greater compared to CKD, reflecting acute kidney damage in AKI patients as compared to chronic damage in CKD due to progressive loss of kidney damage. Moreover, the increasing trend is seen in the further staging of CKD (Fig.1B), which confirms its role in early diagnostic biomarkers. Klotho mitigates inflammation, oxidative stress and fibrosis, regulates the renin-angiotensin-aldosterone system and its restoration offers renoprotective benefits in CKD and AKI.^23^ There is a lack of local research on Klotho in CKD and AKI patients. Devaraj S et al. reported that Klotho level was significantly higher in CKD patients,^24^ which is consistent with our study.

The results of the current study found that the level of B2M was significantly higher in CKD and AKI patients compared to controls (Fig.1), consistent with Sedighi O et al. who revealed that B2M level was elevated in CKD patients. This level progressively increased with decreasing GFR and reported that kidneys eliminate B2M via glomerular filtration and tubular catabolism, due to which B2M is highly correlated with GFR.^25^ There is a lack of local research on B2M in CKD and AKI patients. Cerezo I et al. found that the levels of B2M showed a fair relationship between patients’ mortality and the risk of CKD progression.^26^ Barton KT et al. reported that serum B2M is strongly associated with AKI and showed a graded increase with increasing severity of AKI.^27^ A meta-analysis of 23,318 individuals revealed that creatinine-based and eGFR equations incorporating BTP and B2M indicated declining kidney function with chronic kidney disease progression, especially after adjusting for risk factors, with optimal results from combined eGFR equations.^28^

Our study result demonstrates that serum values of uromodulin were lower in cases than in normal adults (Fig.1A), which indicates its importance as a promising biomarker in early diagnosis of the pathophysiology of the kidney. AKI patients had lower levels of serum uromodulin, reflecting tubo-interstitial kidney damage as compared to chronic/progressive injury in CKD. Moreover, a decreasing trend of serum uromodulin can be seen in patients at different CKD stages. Literature search did not show any local study on uromodulin in CKD and AKI patients. Uromodulin is a biomarker for early CKD and its concentrations decrease with CKD stages,^29^ which is consistent with our study (Fig.1B). Steubl D et al. reported that higher serum uromodulin is independently associated with lower risk for mortality, cardiovascular events and kidney failure in white patients with CKD.^30^ Further, Lv L et al. found serum uromodulin is independently associated with an increased risk of incident ESKD in CKD patients.^31^

The results of the current study found that the level of ADMA was significantly higher in CKD and AKI patients compared to controls (Fig.1). ADMA is the most potent endogenous inhibitor of nitric oxide synthase (NOS), with higher levels in patients with ESRD.^32^ Saran R et al. reported that the level of ADMA was significantly higher in the plasma of CKD patients compared with controls.^33^ Asmarawati TP et al identified levels of ADMA in 3-5 stages of CKD patients and found significant differences in CKD stages, with higher levels indicating increased severity, which is consistent with our study.^34^

Current study results found that the level of NGAL was significantly higher in CKD and AKI patients compared to controls (Fig.1). Shoaib M et al. (2019) showed that the accuracy of urine NGAL was 90.7% in AKI patients.^35^ No study from Pakistan could be found on NGAL for detecting CKD at the national level. However, Abdulameer AN et al. results showed an increase in NGAL level in CKD patients versus controls and revealed a high positive correlation between NGAL and creatinine.^36^ Patel ML et al. reported the progressive increase in NGAL level from CKD stage 2-4, which is consistent with our study.^37^ Consistent with our study, Naqvi R et al. reported NGAL as highly predictable biomarker for AKI.^38^.

Therefore, majority of tested biomarkers show strong potential for reliable CKD detection. In our study, we identified seven promising biomarkers-BTP, Fetuin-A, Klotho, Uromodulin, ADMA, NGAL and B2M-that demonstrated significant diagnostic potential for distinguishing between CKD, AKI and healthy controls (Table-I). Among these, NGAL and ADMA showed exceptional performance, with AUC values of >0.93 in differentiating both CKD and AKI from healthy individuals. Similarly, Klotho, Uromodulin and BTP also exhibited very high AUCs (>0.98) in CKD group, highlighting their potential in early detection markers and these biomarkers show highly effective for the early detection of CKD, particularly when compared with AUC of AKI group Bansal A et al. has reported that abnormal blood levels of BTP, NGAL, kidney injury molecule-1 (KIM-1) and ADMA are associated with abnormal renal function.^39^

Their finding suggests the potential role of these biomarkers in evaluating CKD severity and progression. Although B2M showed slightly lower performance in differentiating AKI from CKD (AUC:0.88000), it maintained excellent accuracy in identifying CKD from healthy individuals (AUC:0.9929) (Table-I). These biomarkers collectively offer a robust diagnostic model that can enhance early detection and disease staging in kidney-related pathologies. In future clinical applications, they may hold potential for personalized risk assessment and prognostic evaluation in reducing the global burden of CKD.

Integrating advanced biomarkers with conventional tests enhances clinical workflows for kidney diseases by providing detailed insights into renal health, allowing for earlier diagnosis and better risk stratification. For instant, NGAL serves as an early diagnostic tool for AKI before creatinine elevation, allowing quicker intervention.^40^ BTP better estimates GFR in specific patients, like those with low muscle mass.^41^ Similarly, Klotho and Fetuin-A offer prognostic insights into CKD progression and vascular calcification but are hindered by research inconsistencies and standardization challenge.^42^ Although it requires further validation. Our study focuses on biomarkers that have been reported in the literature,^8^-^14^ to possess potential of early detection in CKD/AKI by ELISA. ELISA assay cannot claim itself for early diagnostic tool but, our findings demonstrate their association with biomarkers on disease status at the time of sampling. Therefore, our findings have significant implications for the field of nephrology.

However, the broader clinical applicability of many of these markers often confined to research due to various limitations. The lack of assay standardization presents a major challenge, resulting in inconsistent study findings and complicating universal reference ranges for clinical application. Many assays do not isolate kidney-specific markers effectively, making them susceptible to extrarenal influences like inflammation and infection, which may yield false positives. For example, NGAL levels can rise during systemic inflammation,^43^ while B2M is affected by high cell turnover.^44^ The future of these markers lies in multi-marker panels that combine various strengths for a comprehensive kidney health assessment. Improving standardization and cost-effectiveness of multi-marker approaches has transformative potential for kidney disease management, paving the way for better patient outcomes and personalized care.

### Strength of the study:

To the best of our knowledge, this is the first report of its kind from Pakistan to comprehensively evaluate a panel of seven novel biomarkers in differentiating CKD, AKI and healthy individuals, providing valuable insights for local clinical practice. The integration of advance biomarkers, ROC analyses, correlation matrices and STRING network evaluations enhances the robustness of our findings.

### Limitations

The study is limited by its single-center, case-control design and modest sample size, which may restrict generalizability.

## CONCLUSIONS

Study markers show excellent performance (AUC >0.99) for CKD diagnosis, particularly BTP NGAL, B2M, ADMA and uromodulin. These biomarkers correlate with CKD, suggesting their use for early detection, disease staging, and potentially improving CKD diagnosis.

### Authors’ Contribution:

**BF:** Conceived the idea, prepared the manuscript, performed trial screening and identified parameters.

**MSL:** conceived the study idea and performed trial screening, parameter identification and article revision.

**MF and ZHQ:** Literature search, perform trial screening and identify parameters.

All authors have read and approved the final version and are accountable for the integrity of the study.
